# Using a wearable system combining inertial and force sensing for simultaneous detection of limb motion and grasping actions in the workplace

**DOI:** 10.1017/wtc.2025.10026

**Published:** 2025-09-25

**Authors:** Matteo Musso, Shaoping Bai, Anderson Oliveira

**Affiliations:** Department of Materials and Production, Aalborg University, Aalborg, Denmark

**Keywords:** wearable sensors, FSR, IMU, motion tracking, exoskeleton

## Abstract

Using wearable sensors to evaluate workers’ performance is challenging with existing sensor techniques. It requires detecting not only limb motions but also the onset and offset of specific actions. Commonly used inertial measurement units (IMUs) can be combined with surface electromyography (sEMG) to detect muscular activity. However, sEMG requires skin preparation and careful sensor placement, and can be affected by sweat or motion artifacts. To address these limitations, we used a wearable system combining IMUs and force-sensing resistors (FSRs), where IMUs capture joint kinematics and FSRs detect grasping actions. The system included three IMUs (on the trunk, upper arm, and forearm) and two FSR arrays (on the upper and lower arms). The system was first validated in a laboratory setting against an optical motion capture system with 10 healthy young adults performing isolated upper limb movements and mimicking lifting tasks. The results showed high agreement in joint angle estimation (coefficient of multiple correlation = 0.95 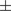 0.04), with a maximum root mean square error of 8.7 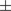 2.92°, and a mean absolute timing error for grasp detection of −0.59 seconds. To evaluate its applicability in real-world scenarios, a pilot in-field test was then conducted with two manufacturing workers (using and not using a passive shoulder exoskeleton) during a repetitive panel-packing task. The test shows highly consistent grasping detection, which allowed segmenting the task with a small variability in task duration (maximum coefficient of variation = 5.16 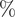 ). These findings demonstrate the feasibility of using the proposed method in industrial environments to analyze upper limb motion and grasping activity.

## 1. Introduction

There has been a growing use of wearable sensors to evaluate workers’ performance and maintain or improve their health, safety, and productivity (Patel et al., 2021). In particular, inertial measurement units (IMUs) have gained popularity among researchers and industry entities seeking to track human motion in real-world working environments (Digo et al., 2022). IMU-based measurements offer a more practical solution for ergonomic assessments in the field compared to gold-standard techniques such as optical motion capture. IMUs require a lower number of sensors to determine body segment motion and do not require large spaces for recordings. Moreover, optical motion capture systems experience marker occlusion, which can compromise motion tracking by causing gaps in segment trajectories. Although IMUs offer several advantages, the technology also presents limitations related to magnetic interferences, which may be amplified in ergonomic settings, as well as the high susceptibility for data drift in long recordings (Digo et al., 2022). Nevertheless, IMUs have been widely used to evaluate human motion under real-world conditions (Digo et al., 2022).

IMU systems have been used for in-field risk assessments, collaborative robotics, motion tracking in industries, action recognition (Digo et al., 2022), and assessing the influence of exoskeletons on human performance (Amandels et al., 2019; Seiferheld et al., 2022). Because IMUs generate only kinematic data, researchers often combine them with physiological or mechanical measures such as surface electromyography (sEMG), spiroergometry systems, and questionnaires to enhance the assessment of physical demands. sEMG, in particular, is used to assess the muscle electrical activity during tasks. sEMG has been used for ergonomic risk score evaluation (Peppoloni et al., 2016; Giannini et al., 2020; Bangaru et al., 2022; Hubaut et al., 2022), human–robot collaboration (Wang et al., 2019; Chico et al., 2021; Lin and Peng, 2022), evaluation of aid provided by new technologies (Amandels et al., 2019; Seiferheld et al., 2022), and activity recognition (Tao et al., 2018; Al-Amin et al., 2019; Kubota et al., 2019; Bangaru et al., 2021). In these studies, sEMG sensors were typically placed around the forearm to detect grasping activities during upper limb movements. However, conventional sEMG measurements require adequate skin preparation, and data quality might be affected by sweat and movement artifacts. Moreover, sEMG sensor placement plays a crucial role in data quality, as signals from adjacent muscles (i.e., crosstalk) can contaminate recordings from the target muscle. Additionally, electrodes may shift due to skin movement during dynamic contractions, leading to erroneous interpretation of the sEMG data (Oliveira and Negro, 2021). As a result, assessing sEMG during real-world ergonomic tasks is highly challenging and often impractical, considering the need for lengthy setup and potentially inaccurate data.

Force-sensing resistors (FSRs) have been explored as an alternative method for estimating muscle activation to address the limitations imposed by surface sEMG. Esposito et al. (2018) reported a strong association between FSR and sEMG signals captured during flexor carpi ulnaris muscle activation during various grasping actions. Additional studies have successfully used FSR bands placed on the forearm to distinguish arm and forearm position and gestures (Xiao and Menon, 2014; Xiao and Menon, 2017a; Islam and Bai, 2020; Rehman et al., 2022), as well as to accurately detect grip and release actions (Wininger et al., 2008; Xiao and Menon, 2017b). In industrial contexts, FSRs have been used in shoe insoles to function as weighing sensors, to detect object picking and drive a back-support exoskeleton accordingly (Mateos, 2017) and monitor the weight of objects carried by construction workers (Lee and Son, 2021). However, FSRs have not yet been used to sense or analyze forearm muscle activation during tasks performed in real work environments.

In this study, we use a wearable sensor system (WSS) that combines IMU and FSR sensors to track upper limb motion in real-world ergonomic settings. The IMU sensors provide kinematic data, while the FSR sensors detect muscle activation in the arm and forearm to identify the onset and offset of movement cycles. The WSS allows simultaneous tracking of upper limb motion and hand actions. We first conducted a controlled laboratory validation to assess the accuracy of the WSS in estimating joint angles and detecting grasping onset and offset. Subsequently, a preliminary in-field test was conducted with two factory workers to examine their motion patterns using the WSS and explore potential differences in movement with and without the use of an exoskeleton.

## 2. IMU/FSR WSS

### 2.1. Sensors

**Figure 1.**
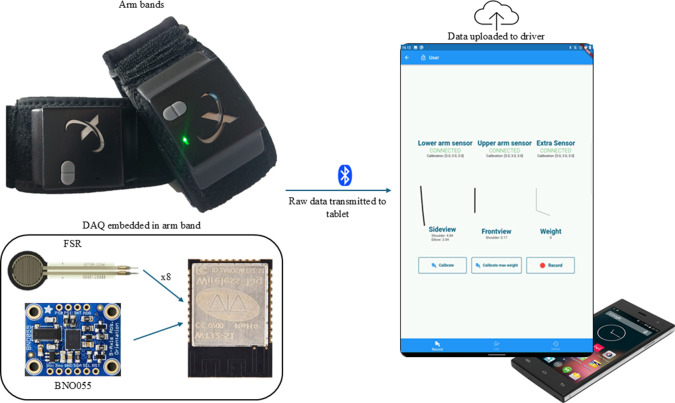
Illustration of the components used to create the WSS. Two BioX armbands containing IMUs and FSRs were used to acquire upper limb movement and muscle activation. The data are transmitted via Bluetooth to a tablet, which saves it to a cloud service and computes real-time joint angles.

The WSS consists of two instrumented armbands (Biox ApS, Aalborg, Denmark) and one trunk-mounted instrumented band. Each of the two armbands and the trunk band includes one IMU (BNO055, Robert Bosch GmbH, Gerlingen, Germany), which contains a gyroscope ( 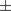 2,000°/s), an accelerometer (up to 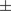 16 G), and a magnetometer ( 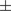 1.3 mT *x* and *y* axes, 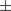 2.5 mT *z* axis). Additionally, each armband contains eight FSRs distributed equidistantly around the band, with a sensing range of 0.2–20 N. Both the armbands and the trunk band are equipped with an ESP32 chip, which acquires data from the IMU and FSR sensors and transmits it wirelessly to a tablet (Tab M10 HD, Lenovo, Beijing, China). An Android application has been developed to establish Bluetooth communication between the ESP32 and the tablet. The application enables real-time acquisition of IMU and FSR data, initial joint angle computation, and data storage on a cloud server at a frequency of 18 Hz. A schematic of the system is shown in Figure 1.

**Figure 2.**
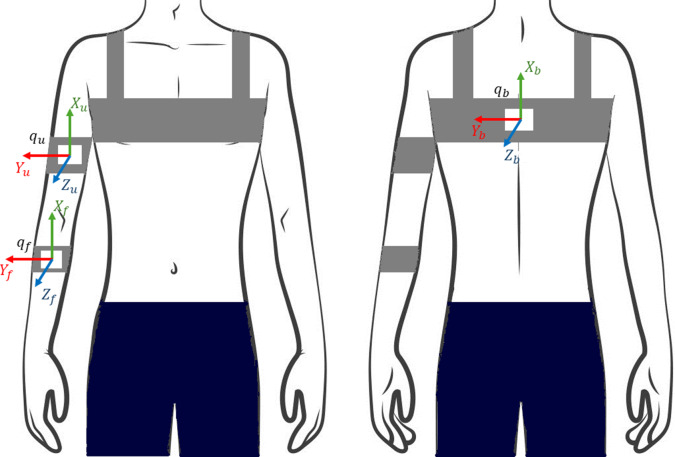
IMU sensor placement and orientation of the sensors’ internal reference systems.

In this study, the armbands were positioned on the anterior sides of the forearm and upper arm, firmly fixed where these segments have the greatest cross-sectional area. We ensured that once placed, the bands did not slip on the arm and did not cause discomfort to the user, but the initial armband tension was not standardized among participants. For the trunk, the IMU was placed between the shoulder blades using a customized, adjustable tank top (Figure 2), with the sensor securely attached.

### 2.2. Estimation of shoulder and elbow joint angles

The internal reference coordinate system of each IMU is defined as follows: the *x*-axis points upward, the *y*-axis is aligned with the human frontal axis, and the *z*-axis is parallel to the sagittal axis. Accordingly, rotations around the *y*-axis correspond to flexion/extension in the sagittal plane, rotations around the *x*-axis correspond to abduction/adduction, and rotations around the *z*-axis correspond to internal/external rotation. The quaternions representing the orientation of each IMU are extracted and used to estimate shoulder and elbow angles using custom scripts (MATLAB R2023a, The MathWorks, Inc., Natick).

To calculate shoulder angles, the quaternion representing the orientation of the back sensor ( 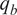 ) is conjugated and multiplied by the quaternion of the upper arm sensor ( 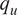 ), to compute the shoulder quaternion ( 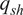 ):.(2.1)

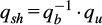



The shoulder angles are then obtained by converting ( 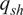 ) into Euler angles using the “YZX” rotation sequence. The angle around the *y*-axis corresponds to flexion/extension (and is negated), while the angle around the *z*-axis corresponds to abduction/adduction.

To estimate elbow angles, the quaternion from the upper arm sensor ( 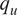 ) is conjugated and multiplied by the forearm sensor quaternion ( 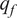 ) to obtain the elbow quaternion ( 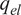 ):.(2.2)

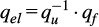



The resulting quaternion 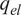 is then converted to joint angles using the “YZX” rotation sequence. No negation of the *y*-axis angle is needed as both sensors’ axes are aligned with the same orientation.

## 3. Laboratory validation test

The WSS was tested on 10 healthy young volunteers (nine males, one female; 28.2 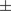 3 years; height 179.15 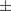 7 cm; body mass 78.9 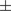 14 kg). The exclusion criteria included any impediments to arm motion and/or pain in the upper limb joints. All participants received written and verbal instructions about the experiment and provided written informed consent. The experimental procedures were approved by the local ethics committee (case number 2024-505-00159).

### 3.1. Experimental design

Participants were initially equipped with two armbands on the right arm and the trunk-mounted tank top as described in Section 2.2. A tablet was used to verify the initial alignment of the IMU sensors and ensure data quality. Retroreflective markers were subsequently attached for simultaneous optical motion capture using a 12-camera system (Qualisys AB, Göteborg, Sweden; 100 Hz sampling frequency). The Plug-in Gait Full-Body model (Vicon, 2024) was used to define the trunk, right forearm, and upper arm segments based on 11 retroreflective markers. Subsequently, a musculoskeletal model was created using the marker trajectory data in Visual3D (HAS-Motion, Kingston, ON, Canada) to replicate the trunk and right forearm, upper arm segments (Has-Motion, 2024). Before experimental trials, a single 5-second recording of each participant in neutral reference posture (N-pose) was performed to scale the Visual3D model to the participants’ size.

Participants performed the following upper limb movements:

T1 Shoulder flexion (from 0 to 90°) in the sagittal plane.

T2 Shoulder flexion (from 0 to 90°) at a 45° angle between the sagittal and frontal planes.

T3 Shoulder abduction (from 0 to 90°).

T4 Elbow flexion (from 0 to 90°) in the sagittal plane.

T5 Combined shoulder and elbow flexion/extension in the sagittal plane. Participants were asked to first flex their elbows from 0 to 90°, then performed a shoulder flexion from 0 to 90° while maintaining the 90° elbow angle. Subsequently, the participant extended the elbow to 0° and then the shoulder back to 0°.

T6 Shoulder flexion (from 0 to 160°) in the sagittal plane, performed with the torso flexed approximately 40° forward.

T7 Simulated work task: Participants simulated picking up an object from the ground, walking 6 m, placing it down, and standing upright for 2 seconds. The participants did not have an actual object. After 2 seconds, the task was repeated. During walking, participants were instructed to slightly face a side wall, as if carrying the object with another person. This sequence was repeated 10 times.

**Figure 3.**
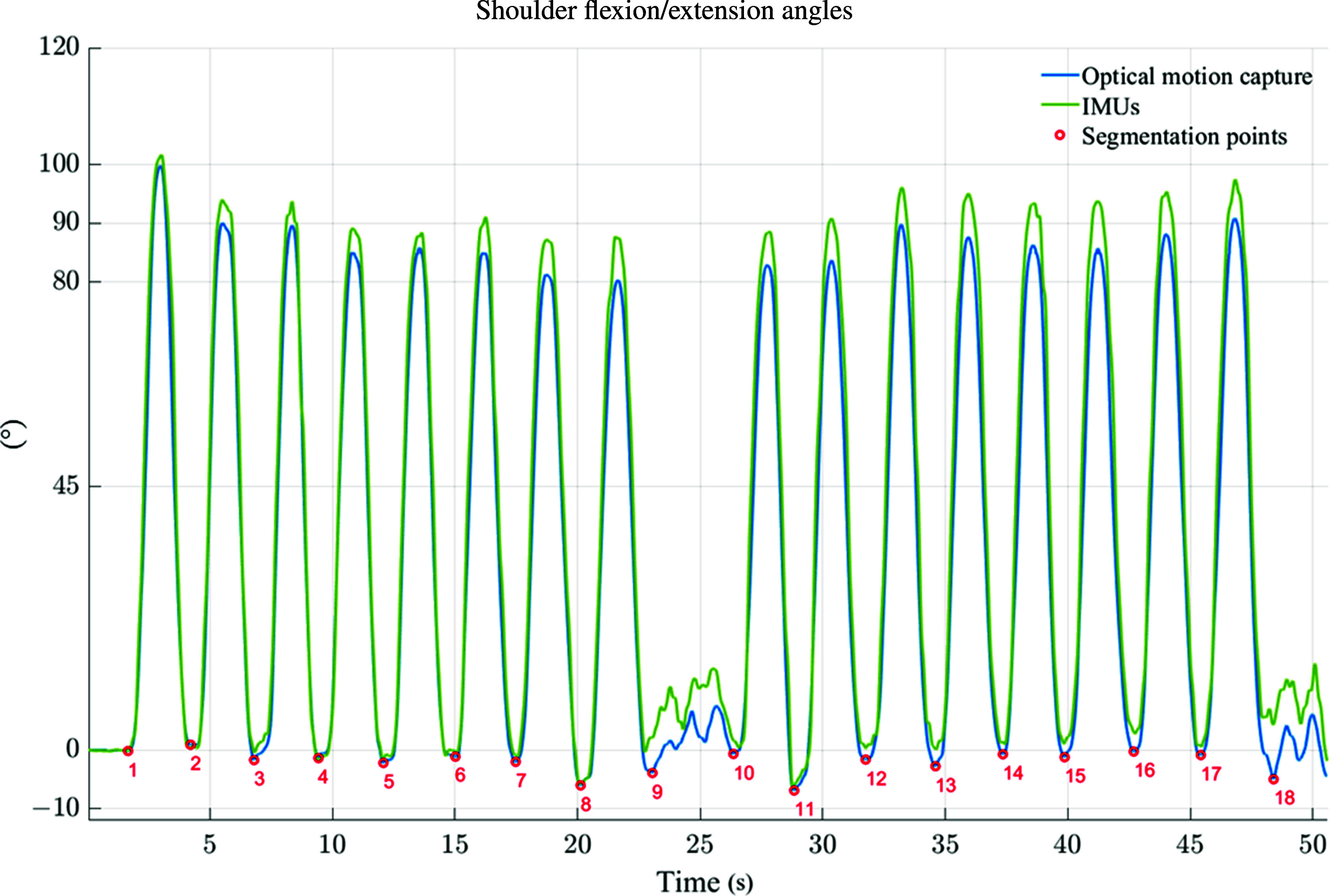
Computed shoulder flexion/extension angles from the two methods and segmentation results.

Participants performed all tasks at a self-selected speed. Tasks T1–T6 were repeated in four sets of eight repetitions. After each set, participants were asked to move and rotate around the vertical axis to change the sensor orientation, allowing us to evaluate the versatility of the motion-tracking system in estimating joint angles. Figure 3 shows sample shoulder flexion/extension angles derived from both the optical motion capture and the IMU system.

### 3.2. Data processing and analysis

#### 3.2.1. IMU data

All data were processed using custom-made MATLAB scripts (The MathWorks, Inc., Natick). The tridimensional optical marker position data were labeled, gap-filled (Qualisys Track Manager 2023.1, Qualisys, Göteborg, Sweden), and exported as C3D files. The subjects’ Visual3D models were created from N-pose C3D files. Visual3D models were created from the N-pose recordings and then applied to movement tasks to extract shoulder and elbow joint angles.

IMU-derived joint angles were upsampled to 100 Hz using MATLAB’s resample function (default linear method) to match the sampling frequency of the optical motion capture data and synchronized using cross-correlation. Shoulder and elbow joint angles from the optical motion capture system were segmented by identifying the minimum point immediately before or after the maximum flexion angle of each task repetition. These segmentation points were used to define movement cycles in the joint angle data of both the optical motion capture system and the WSS (Figure 3).

The WSS’s accuracy in tracking upper limb kinematics was evaluated using the coefficient of multiple correlation (CMC) (Ferrari et al., 2010), where values between 0.75 and 0.84 indicate good, 0.85 and 0.94 very good, and 0.95 and 1 excellent agreement (Ferrari et al., 2010; Zhang et al., 2013). In addition, the absolute (RMSE) and relative root mean square error (RRMSE) were also calculated (Ren et al., 2008). CMC, RMSE, and RRMSE were computed for each movement cycle and then averaged per participant and task. The 95% confidence interval and standard deviation were calculated. Finally, the agreement between the two measurement systems was assessed using Bland–Altman plots (Bland and Altman, 1986), a commonly used method for comparing the performance of a new technology against that of the gold standard one (Mihcin, 2019, 2022). The maximum and minimum joint angles during each task repetition, obtained from both methods, were compared.

#### 3.2.2. FSR data

**Figure 4.**
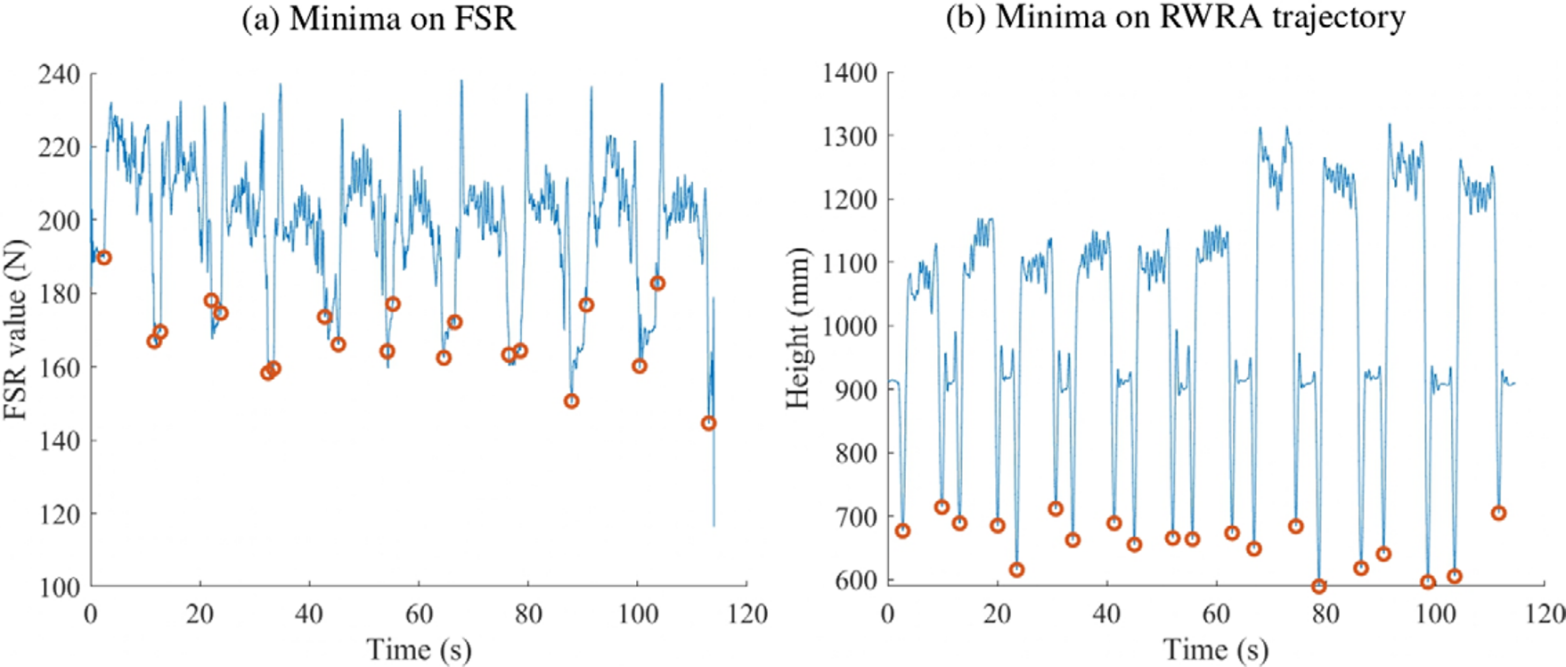
Identified minima (red circles) in the average FSR signal (a) and RWRA marker trajectory (b) during the simulated work task.

To evaluate the use of the FSRs for identifying grasping and releasing events, the simulated work task recordings from the optical system were segmented by identifying the minima in the vertical position of the right wrist marker A (RWRA; Figure 4). These minima corresponded to the box grasping and releasing events and were therefore used to define complete task cycles. The eight FSR signals from the forearm band were averaged and segmented based on the minima occurring before and after signal bursts. We used the averaged signal due to the simplicity of the target movement. The grasping and lifting movement involved all the fingers as well as the wrist, and therefore strongly engaged the forearm muscles. For more complex movement, a classification algorithm or spatial analysis would probably be required. These bursts were detectable due to increased activation of wrist and finger flexor muscles when the box was picked up and moved during the task. The minima delimiting each burst were considered the start and end points of the grasping actions. We selected the minima as they indicate no pressure on the hand and hand open, corresponding to the minimum recruitment of the forearm muscles. Using the burst peak values to identify grasping would likely be more indicative of the lifting phase, when the full weight of the box is supported by the hand, rather than the grasping action itself. The timing of wrist marker minima was then compared with the corresponding FSR minima to assess the accuracy of grasp/release event detection. The average absolute and relative errors across all 10 participants were reported separately for grasping and releasing actions.

### 3.3. Validation results

#### 3.3.1. Limb motion tracking

**Figure 5.**
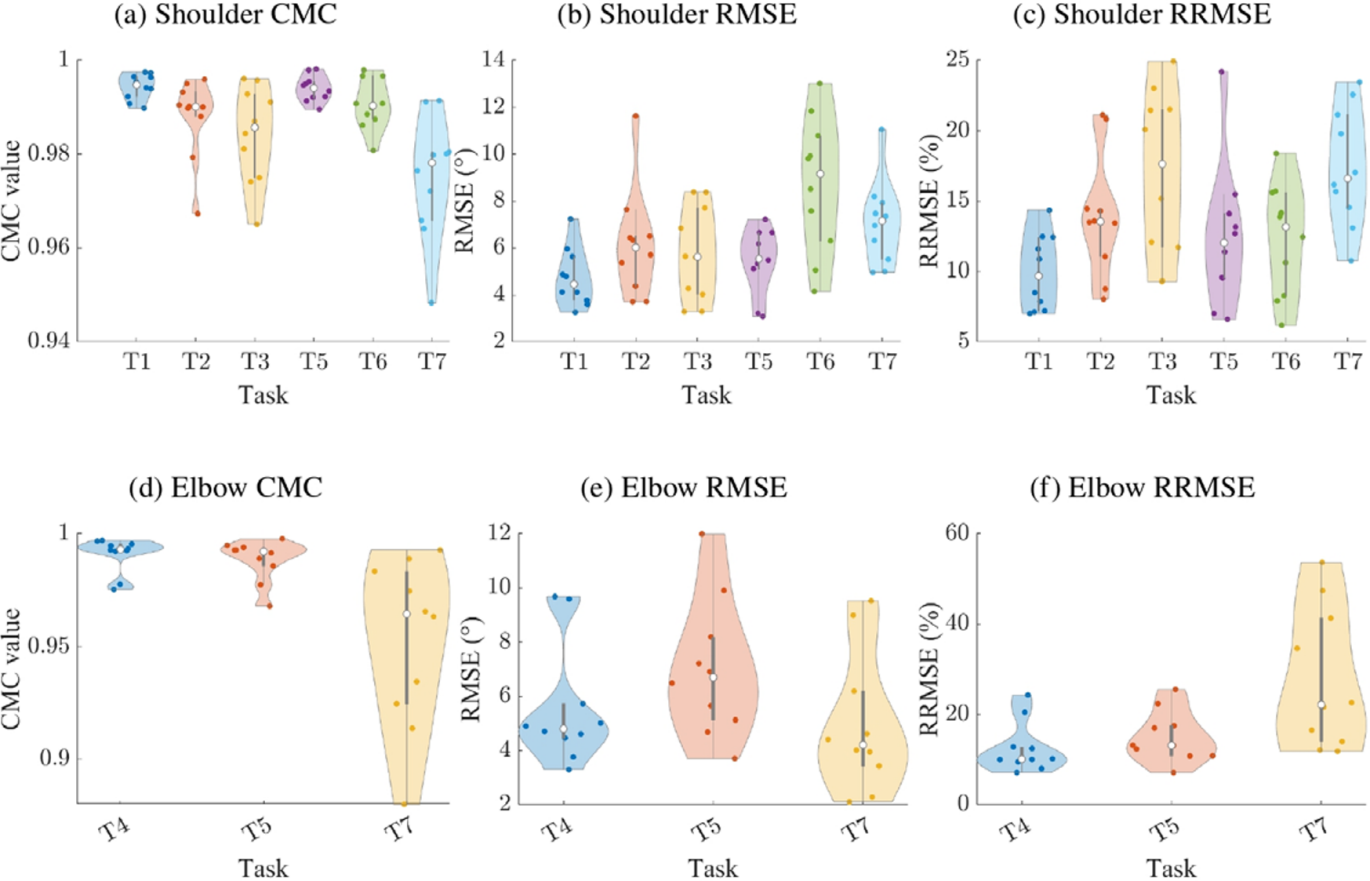
Violin plots of the coefficient of multiple correlation (CMC; a and d), root mean square error (RMSE; b and e), and relative root mean square error (RRMSE; c and f) computed from the comparison between optical motion capture and IMU-derived joint angle calculations for the shoulder (upper row) and elbow (bottom row). Each colored dot in a violin represents the average value for one participant, the white dot represents the median across participants, the gray vertical bar indicates the first and third quartiles, and the shaded colored area depicts the density curve. The tasks are described in Section 3.1.

**Figure 6.**
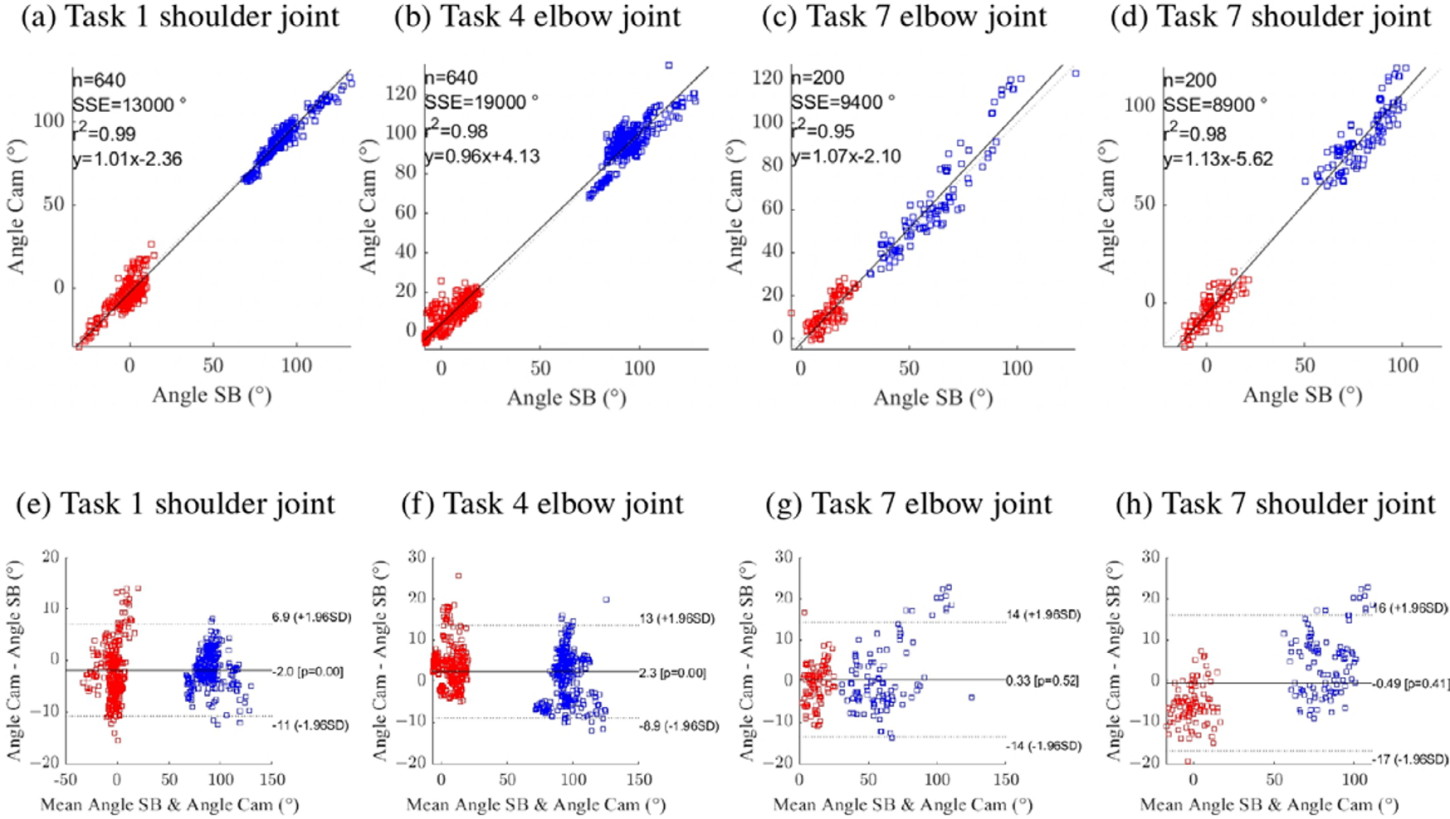
Correlation and Bland–Altman plots for three selected tasks: T1, T4, and T7.

The joint angles estimated using the WSS showed excellent agreement with those obtained from the gold standard, as indicated by high CMC values for both the shoulder (Figure 5a) and elbow joints (Figure 5d) across all isolated tasks. RMSE values remained below 10° across all tasks and joints. For the shoulder, the highest RMSE and intersubject variability were observed in tasks T2 and T6 (Figure 5b). Similarly, the highest RMSE for the elbow was observed during task T5 (Figure 5e). RRMSE ranged from 7 to 25% for the shoulder (Figure 5c), with consistent patterns across tasks. The average RRMSE exceeded 16% in two tasks: T3 (16.9 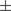 5.98 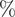 ) and T7 (16.3 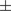 3.87 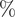 ). For the elbow (Figure 5f), the RRMSE was notably higher during T7. A summary table of the CMC, RMSE, and RRMSE values is provided in Supplementary Table S1. Figure 6 and Supplementary Figure S1 present the correlation and the Bland–Altman plots for shoulder and elbow angles. The Bland–Altman plots also report the mean difference and limits of agreement. The largest mean difference was observed during task T2 on the shoulder, with an absolute value of 3.9°. The smallest was 0.33° during task T7 on the elbow.

#### 3.3.2. Grasping action recognition

For the grasping events, the average absolute and relative errors between the timings of the identified minima in the FSR average signals and the RWRA trajectory were −0.59 seconds and −4.39%, respectively. For releasing events, the values were 0.54 seconds and 1.49%.

## 4. In-field test

Following laboratory validation of the WSS, in-field testing was conducted to demonstrate the feasibility of the new technology. The field tests were conducted at a company producing kitchen furniture. Two females (32 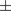 12 years, height 171.5 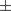 2 cm, body mass 63 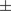 1 kg) participated in the field test. We evaluated the WSS’s sensitivity in detecting changes in motion patterns when workers performed their tasks with and without an exoskeleton.

**Figure 7.**
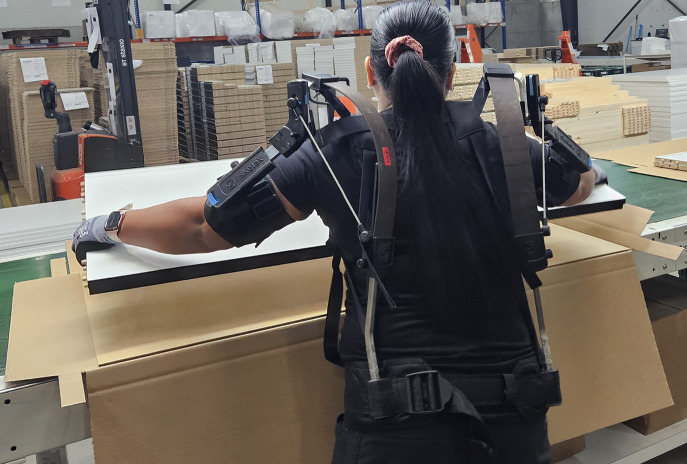
Illustrative image of a worker performing the task of moving a panel for packing. The workers performed the packing task with and without using a passive exoskeleton (Skelex 360-XFR). The task involved shoulder abduction and flexion, combined with elbow flexion. WSS was used to identify the grasping moments for subsequent segmentation of joint angles.

The workers were responsible for packing kitchen cabinet parts. Worker 1 was responsible for packing four 2.4 kg panels together, while worker 2 was responsible for packing two 4.67 kg panels (Figure 7). Both picked panels from a stack behind them and placed them in pairs into a box, with side tasks such as placing the box on a conveyor belt and adding a thinner panel. The study was conducted in conjunction with a pilot trial to evaluate the feasibility of using a specific passive exoskeleton (Skelex 360-XFR, Rotterdam, the Netherlands, weight: 2.3 kg, support between 0.5 kg and 4 kg/arm) to assist the workers. This test followed a laboratory test evaluating support provided by the device in a similar manual handling activity (Musso et al., 2024). Therefore, we assessed the motion patterns of the two workers during the packing task with (“Exo” condition) and without (“Free” condition) the use of the passive exoskeleton, using the WSS.

**Figure 8.**
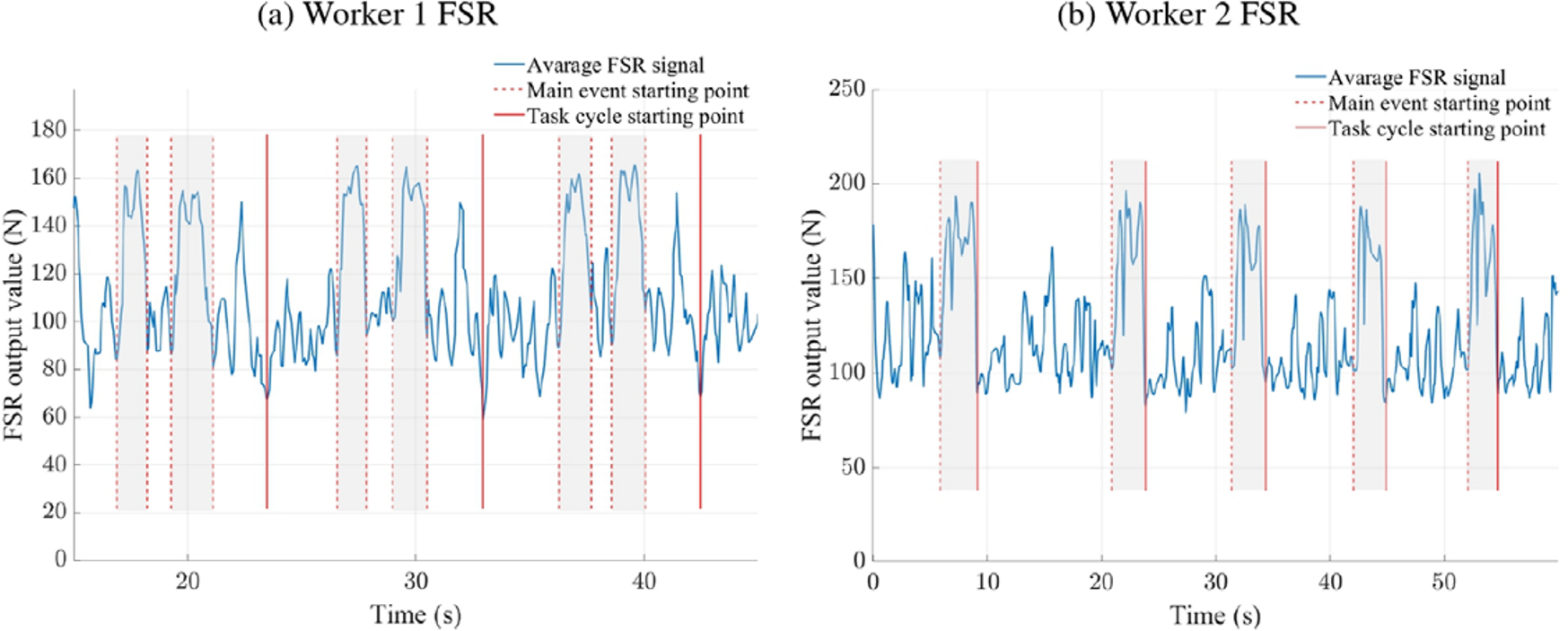
Averaged force-sensing resistor (FSR) signals from the two workers and the identified segments. Dashed lines indicate the start and end of the main events; solid lines indicate the limits of full task cycles. For worker 2, these two sets of lines coincide.

We used the WSS to track the workers’ movements and identify the start and end of each packing cycle. For worker 2, a full cycle was defined as the part of the motion between two consecutive panel packings. For worker 1, it was the interval between packing the thinner panels, which she placed after the main four panels as the final step of her work cycle. Within each cycle, the key event of interest was the panel-packing action, which began when a pair of panels was grabbed from the stack and ended when they were placed on the conveyor belt. This motion involved sequential shoulder and elbow flexion and extension. The signals from the forearm FSR were used to detect grasp and release moments, which were detectable due to the increase in activation of the wrist and finger flexor muscles (see Figure 8 for illustration). Only cycles without missing data points were included in the analysis.

The start and end times were used to segment joint angle and angular velocity time series. The angular velocity of the arm IMUs around their internal *y*-axes was interpreted as the flexion velocity of the proximal joint. Worker 1 performed 32 movement cycles (16 for the Free condition and 16 for the Exo condition), while worker 2 performed 53 movement cycles (21 for the Free condition and 32 for the Exo condition). The cycles under the same conditions were not all recorded together but in up to 3 subsequent sessions. Subsequently, data from each cycle were time-normalized to 80 points, representing 0–100% of the cycle. Subsequently, a median representative curve was generated from the multiple cycles for each worker. A median curve was generated for each worker, and cycles with a correlation coefficient <0.6 with the median were excluded to remove outliers and non-standard movements, such as discarding a defective panel.

For each cycle, the main event duration, total cycle duration, shoulder and elbow range of motion (ROM), and both average and peak angular velocities were calculated for the Free and Exo conditions. The coefficient of variation (CV = 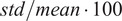 ) was also calculated for some parameters to evaluate segmentation consistency.

Due to the small sample size, kinematic results are reported separately for the two workers (Table 1). For worker 1, 12 cycles were included under the Free condition and 14 under the Exo condition. For worker 2, 19 Free and 28 Exo cycles were analyzed.

**Table 1.** Summary of the movement cycle parameters for the two workers under the two conditions, along with the percentage differences between the “Free” and “Exo” conditions

	Worker 1			Worker 2		
	Free	Exo	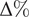	Free	Exo	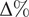
Main event duration (s)	2.2 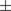 0.56	2.9 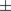 0.43	+35.09%	3.7 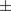 0.49	3.7 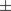 0.61	+1.456%
Cycle duration (s)	9.4 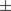 0.28	10 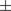 0.3	+8.483%	9.1 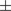 0.37	10 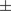 0.51	+9.643%
Shoulder ROM (°)	[90.49 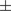 6.5–13.22 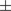 4.8]	[75.8 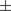 21–9.016 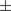 5.2]	[−16.24% +31.79%]	[73.28 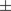 9.2–16.89 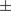 12]	[93.21 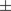 22–2.841 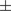 5.8]	[+27.2% +83.18%]
Elbow ROM (°)	[108.9 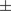 12 6.723 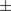 7.5]	[82.91 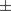 7.8–0.3511 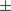 5.7]	[−23.89% −105.2%]	[78.58 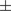 6.2 8.994 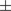 8.4]	[82.52 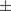 16 2.929 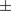 7.8]	[+5.021% −67.43%]
Shoulder flexion velocity (°/s)	86.81 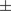 7	73.94 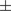 6.4	−14.83%	49.2 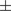 4.2	50.43 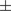 6.8	+2.485%
Elbow flexion velocity (°/s)	73.85 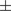 6.8	86.34 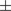 8	+16.91%	45.31 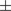 3.8	43.98 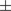 4.7	−2.935%
Shoulder flexion peak velocity (°/s)	302.4 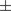 35	250.7 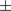 44	−17.07%	171.6 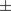 29	162.6 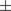 32	−5.258%
Elbow flexion peak velocity (°/s)	263.4 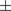 23	320.5 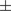 44	+21.65%	215.9 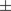 19	176.1 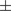 21	−18.46%

*Note*: Results for each condition are reported as the average value 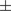 standard deviation. Range of motion results are presented as the average maximum and minimum shoulder flexion angles.

The main event and cycle durations were longer for worker 1 when using the exoskeleton. Additionally, both shoulder and elbow ROM decreased by at least 19% with the exoskeleton. Worker 1’s average shoulder flexion velocity decreased by −15%, while the average elbow velocity increased by −17% when using the exoskeleton. In contrast, worker 2 showed minimal differences (<3%) in average angular velocities between conditions. Regarding peak velocities, worker 1 showed decreased shoulder and increased elbow flexion velocities (−17% and +21%), while worker 2 had a 5% increase in peak shoulder flexion velocity and an 18% reduction in peak elbow flexion velocity.

## 5. Discussion

This study demonstrated that a WSS based on IMUs and FSRs provides accurate measurements of upper limb kinematics and detection of grasping onsets and offsets. Altogether, our findings support the feasibility of using the IMU/FSR WSS, highlighting the potential of such systems for in-field monitoring of movement patterns aimed at reducing the risk of work-related musculoskeletal disorders.

We observed excellent agreement between the joint angle time series from the WSS and the gold-standard optical system, with CMC values exceeding 0.95 across all tasks and joints. The average RMSE across all tasks was approximately 6°, with the highest RMSE being 8.7 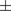 2.92° for task T6. These results are consistent with those reported for other IMU systems, as reviewed in Fang et al. (2023). Previous studies have reported RMSE values between 5° and 10° during various movements (Bouvier et al., 2015; Serra-Hsu and Taboga, 2022; Wu et al., 2022; Zhu et al., 2022). RMSE values exceeding 10° have also been reported (Pérez et al., 2010; Bouvier et al., 2015; Bessone et al., 2022; Henschke et al., 2022) when validating IMU-based systems for joint angle estimation during isolated shoulder flexion/extension or abduction movements. In more complex movements such as box lifting (Robert-Lachaine et al., 2017; Robert-Lachaine et al., 2020; Humadi et al., 2021), shoulder flexion RMSEs up to 35.8° have been reported (Robert-Lachaine et al., 2017), whereas we observed substantially lower errors in similar conditions (RMSE 7°). For elbow flexion, our RMSE results align with previous studies, which reported approximately 6° RMSE (El-Gohary and McNames, 2012; Pérez et al., 2010; Z. Q. Zhang et al., 2011). Our system shows smaller RMSEs than those reported in (Bouvier et al., 2015; Robert-Lachaine et al., 2020; Bessone et al., 2022) for similar movements. Therefore, the proposed WSS demonstrates comparable or superior joint angle estimation performance relative to previously reported systems for upper limb kinematic assessment.

These results were achieved without using a sensor-to-segment calibration step to establish a relationship between the IMU and the anatomical reference system. We ensured that the user maintained the arms along the body and hand in a neutral position while checking the sensor alignment in the tablet, which displayed shoulder and elbow angles close to 0°. A similar approach was used by Chan et al. (2022), who achieved RMSE values comparable to ours. Nevertheless, more accurate results might be achievable using sensor-to-segment calibration, as shown by studies that reported RMSEs below 1° during shoulder movements using MT9B (Cutti et al., 2008) and MARG sensors (Madrigal et al., 2016).

Another factor that may have affected our results is the soft tissue artifact. It refers to the different movements of the markers relative to the underlying bones due to the presence of soft tissues in between (Mihcin, 2019).

The Bland–Altman plots show good agreement between systems. Table 2 shows the percentage of points in every Bland–Altman within the upper and lower limits of agreement (mean 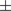 1.96 SD of the difference). According to Bland and Altman (1986), 95% of the data points should fall within these limits. This threshold was reached for tasks 1 and 2; for the remaining tasks, percentages ranged from 91.3 to 94.7%.

**Table 2.** Percentage of the data in the Bland–Altman plots falling within the upper and lower limits of agreement (mean 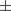 1.96 SD of difference)

	Task						
	1	2	3	4	5	6	7
Shoulder	95%	95.8%	93.8%	N/A	94.7%	93.1%	93.5%
Elbow	N/A	N/A	N/A	94.7%	91.3%	N/A	93%

Previous studies have used full-body IMU systems to evaluate ergonomic tasks (Skals et al., 2021; Seiferheld et al., 2022), but this technology is often costly and requires the handling of numerous sensors. Therefore, further development of simplified WSS approaches is needed to facilitate motion tracking in real-world ergonomic applications.

**Figure 9.**
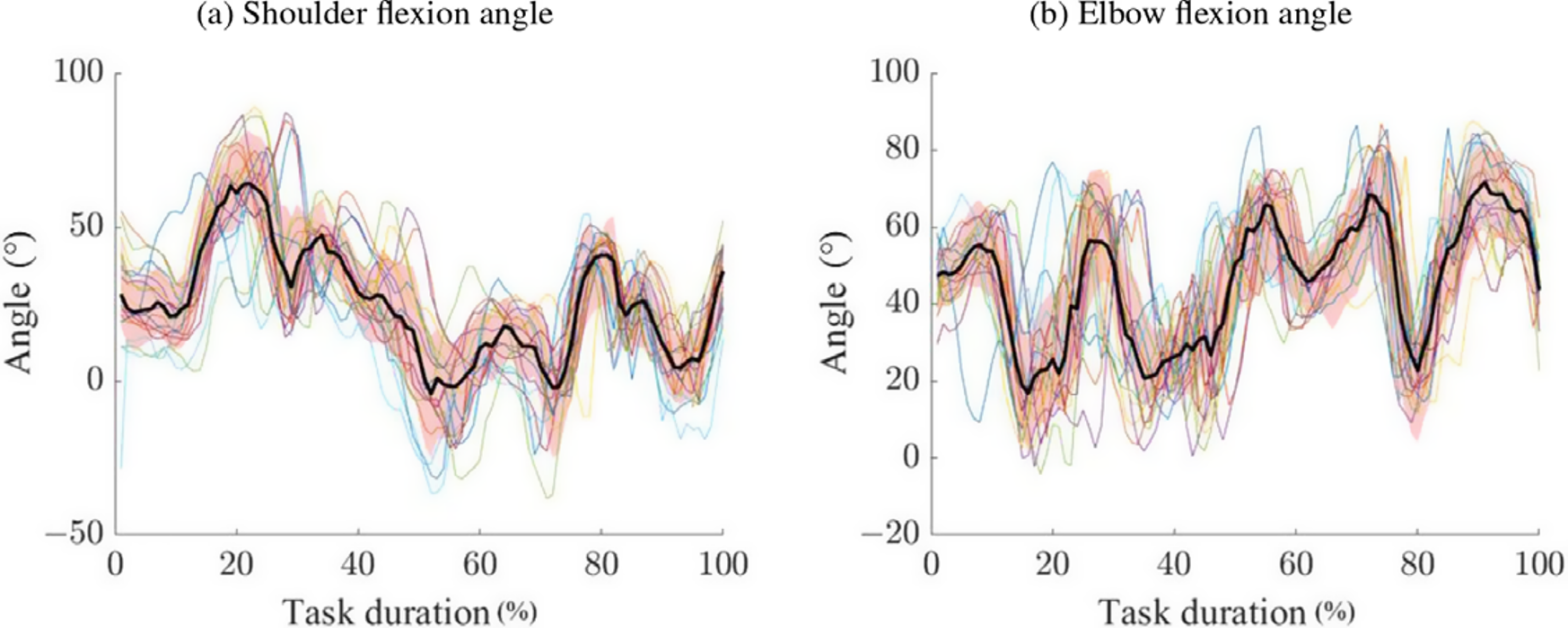
Worker 2 shoulder and flexion angles during full cycles.

The FSR component of the WSS successfully detected grasp and release events during panel handling, with both workers exhibiting consistent joint angles and low inter-trial variability throughout the task cycle (see Figure 9). Considering the full cycle duration, the highest CV was 5.16 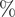 for worker 2 under the Exo condition, while the lowest was 2.96 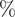 for worker 1 under the Free condition. The consistency of shoulder flexion kinematics across repetitions confirms the potential of the WSS to monitor task execution and identify specific task-related events. The use of an exoskeleton slightly changed the duration of the task ( 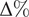 < 10% for both workers). The differences in ROM might have been influenced more by the change in panel stack height during task performance than by the use of the exoskeleton.

The WSS proved easy to handle. Fitting and setting up the sensor bands required only a few minutes per worker and did not interfere with their regular work pace. Moreover, the workers did not report any discomfort when using the WSS throughout their working routines. Similarly, they experienced no issues using the wearable sensor system in combination with assistive tools, in particular, the upper limb exoskeleton in this study. The exoskeleton cuff was placed around the upper arm band, with ample space to fix both devices. This suggests that the WSS can be used for in-field testing of exoskeleton technologies. The limited sample size and the specific task performed in this study limit the generalization of the results obtained. Moreover, our in-field testing was performed only by females, further limiting the generalization of the presented results. Sensor placement in males and females may slightly differ due to anthropometric characteristics, especially around the torso area. Therefore, future studies comprising a larger sample size and the inclusion of a balanced gender distribution will be crucial to establish the system’s applicability and fully validate it for movement analysis in the industrial field.

In addition to the scope of this study, the WSS could be applied to risk assessments Tahir et al., 2023) using tools like the Rapid Upper Limb Assessment (McAtamney and Corlett, 1993), which evaluates joint postures and load handling for ergonomic risk scoring. Another potential application is in human–robot interaction, such as adjusting the level of an exoskeleton based on shoulder angle and object manipulation. This approach is similar to that described in Mateos (2017), but it also includes information on joint angles. Nonetheless, applications beyond those tested here, such as fast-paced movements in sports or tasks requiring more precise estimation of joint angles or grasping timing, will require further validation as the average errors obtained (RMSE = 8.7° and 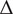 *t* = 0.5 s) may not be sufficiently small. Field testing also revealed occasional data transmission issues from the WSS to the tablet, likely due to Bluetooth interference. Future updates to the Bluetooth module could address this limitation.

## 6. Conclusions

In this work, we developed a wearable motion tracking system that combines inertial and force sensing to simultaneously estimate upper limb motion and detect grasping events. Laboratory-based validation demonstrated strong agreement and satisfactory accuracy for shoulder and elbow kinematics. Moreover, the system identified grasping and releasing actions with a consistent timing error. Finally, the in-field test illustrated the feasibility of using the motion tracking system under real-world conditions by comparing workers’ performance with and without exoskeletons. Future studies should focus on improving joint angle estimation through sensor-to-segment calibration and expanding the system’s application in the human–robot interaction and in-field risk assessment. Evaluating the system in different industrial settings and with more subjects will also be necessary to fully validate it for use in the industrial field.

## Supporting information

Musso et al. supplementary material Musso et al. supplementary material

## Data Availability

Data can be made available to interested researchers upon request by email to the corresponding author.

## Supplementary material

The supplementary material for this article can be found at http://doi.org/10.1017/wtc.2025.10026.
