# Supplementary material for: Using a wearable system combining inertial and force sensing for simultaneous detection of limb motion and grasping actions in the workplace
Source: Wearable Technol. 2025 Sep 25;6:e48. doi: 10.1017/wtc.2025.10026 (PMC12501534; doi:10.1017/wtc.2025.10026)
Supplement: Musso et al. supplementary material [file S2631717625100261sup001.pdf]

Using a wearable system combining inertial and force sensing for simultaneous detection of limb motion and hand actions in the workplace

Matteo Musso, Shaoping Bai and Anderson Souza Oliveira

August 13, 2025

1 Summary table and additional plots of laboratory test results

Table 1 summarizes the CMC, RMSE, and RRMSE data obtained from the laboratory tests.

Table 1: Laboratory test results summarize.

Test	CMC sh	RMSE sh	RRMSE sh	CMC el	RMSE el	RRMSE el
**Shoulder flexion in sagittal plane**	0.99 ± 0.003 [0.99 1]	4.75 ± 1.24° [3.86 5.63]	9.95 ± 2.71%	N/A	N/A	N/A
**Shoulder flexion 45° out of sagittal plane**	0.99 ± 0.009 [0.98 0.99]	6.15 ± 2.32° [4.49 7.81]	13.9 ± 4.35%	N/A	N/A	N/A
**Shoulder flexion in frontal plane**	0.98 ± 0.01 [0.98 0.99]	5.75 ± 2° [4.32 7.18]	16.9 ± 5.98%	N/A	N/A	N/A
**Elbow flexion**	N/A	N/A	N/A	0.99 ± 0.008 [0.99 1]	5.57 ± 2.24° [3.97 7.18]	12.5 ± 5.57%
**Shoulder and elbow flexion combined**	0.99 ± 0.003 [0.99 1]	5.46 ± 1.39° [4.47 6.45]	12.4 ± 5.08%	0.99 ± 0.009 [0.98 1]	6.99 ± 2.51° [5.19 8.78]	15 ± 5.651%
**Shoulder flexion with torso flexion**	0.99 ± 0.005 [0.99 0.99]	8.7 ± 2.92° [6.62 10.8]	12.3 ± 3.96%	N/A	N/A	N/A
**Work task simulation**	0.98 ± 0.01 [0.97 0.98]	7.08 ± 1.82° [5.78 8.39]	16.3 ± 3.87%	0.95 ± 0.04 [0.93 0.98]	4.95 ± 2.56° [3.12 6.78]	27.15 ± 15.5%

Average CMC, RMSE, and RRMSE values for the different tasks and joints. Results for the shoulder are reported as 'CMC/RMSE/RRMSE sh', and those for the elbow as 'CMC/RMSE/RRMSE el'. CMC and RMSE are reported as mean ± SD [95% CI], and RRMSE as mean ± SD.

Figure 1 shows the correlation and Bland–Altman plots for the tasks not included in the main document.

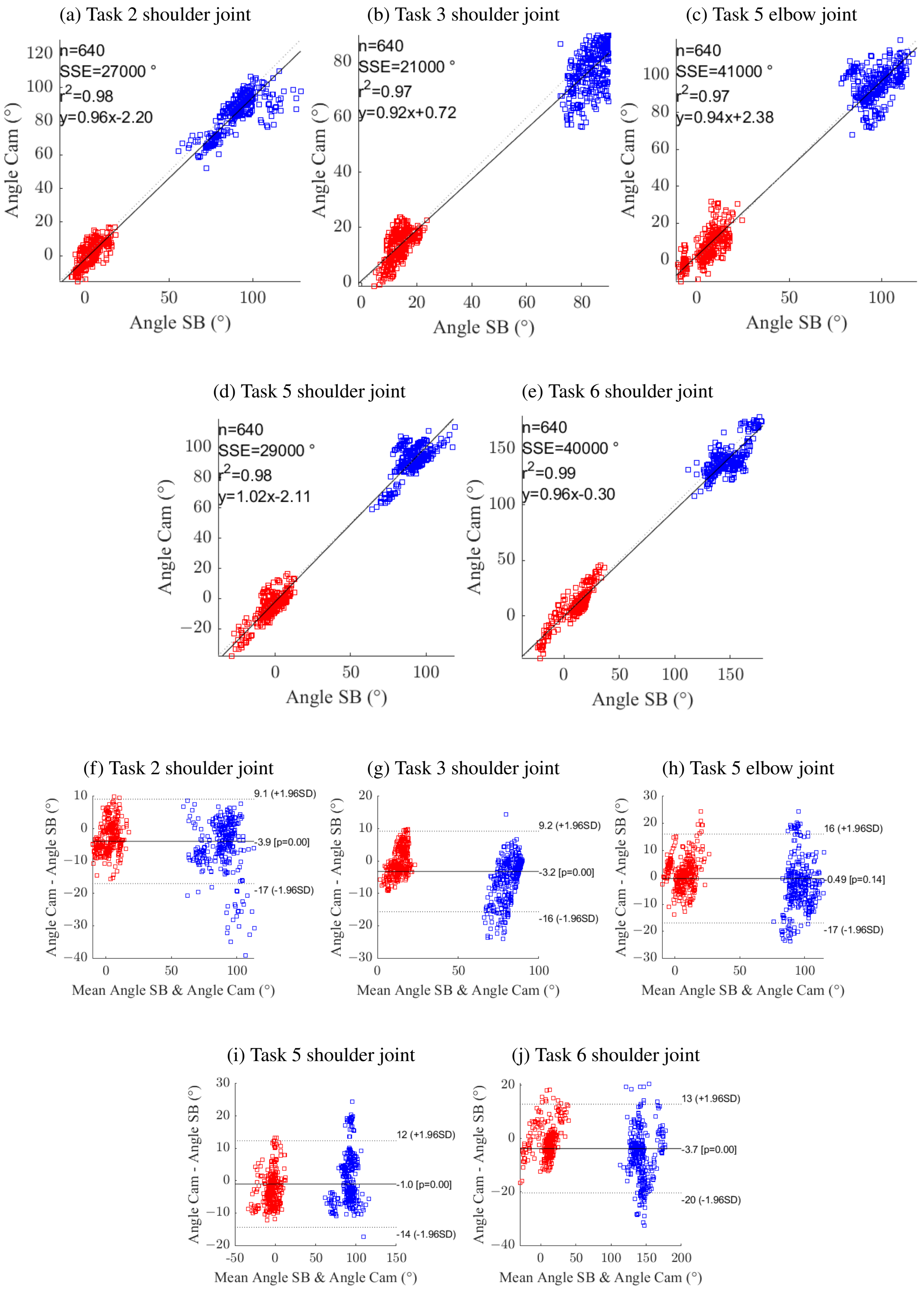

Figure 1: Correlation and Bland–Altman plots for tasks not included in Figure 6 of the main document: T2 – Shoulder flexion at a 45° angle relative to the sagittal plane; T3 – Shoulder abduction; T5 – Combined shoulder and elbow flexion/extension in the sagittal plane; T6 – Shoulder flexion in the sagittal plane with the torso flexed to approximately 40°.
